# Room-temperature serial crystallography using a kinetically optimized microfluidic device for protein crystallization and on-chip X-ray diffraction

**DOI:** 10.1107/S2052252514016960

**Published:** 2014-08-25

**Authors:** Michael Heymann, Achini Opthalage, Jennifer L. Wierman, Sathish Akella, Doletha M. E. Szebenyi, Sol M. Gruner, Seth Fraden

**Affiliations:** a Graduate Program in Biophysics and Structural Biology, Brandeis University, 415 South Street, Waltham, MA 02454, USA; b Martin Fisher School of Physics, Brandeis University, 415 South Street, Waltham, MA 02454, USA; c Field of Biophysics, Cornell University, Ithaca, NY 14853, USA; d Cornell High Energy Synchrotron Source (CHESS) and Macromolecular Diffraction Facility at CHESS (MacCHESS), Cornell University, Ithaca, NY 14853, USA; e Department of Physics, Cornell University, Ithaca, NY 14853, USA; f Kavli Institute at Cornell for Nanoscale Science, Cornell University, Ithaca, NY 14853, USA

**Keywords:** protein crystallization, X-ray diffraction, serial crystallography, microfluidic devices

## Abstract

An emulsion-based serial crystallographic technology has been developed, in which nanolitre-sized droplets of protein solution are encapsulated in oil and stabilized by surfactant. Once the first crystal in a drop is nucleated, the small volume generates a negative feedback mechanism that lowers the supersaturation. This mechanism is exploited to produce one crystal per drop. Diffraction data are measured, one crystal at a time, from a series of room-temperature crystals stored on an X-ray semi-transparent microfluidic chip, and a 93% complete data set is obtained by merging single diffraction frames taken from different unoriented crystals. As proof of concept, the structure of glucose isomerase was solved to 2.1 Å, demonstrating the feasibility of high-throughput serial X-ray crystallography using synchrotron radiation.

## Abstract 2

An emulsion-based serial crystallographic technology has been developed, in which single crystals are grown in nanolitre-sized droplets inside an X-ray semi-transparent microfluidic chip exploiting a negative feedback mechanism. Diffraction data are measured, one crystal at a time, from a series of room-temperature crystals stored in the chip, and a 93% complete data set is obtained by merging single diffraction frames taken from different unoriented crystals to solve the structure of glucose isomerase to 2.1 Å.

## 1. Introduction   

In conventional protein X-ray crystallography, a complete data set is ideally obtained from a single crystal, which typically requires a relatively large crystal that has been successfully cryocooled. Serial crystallography takes the opposite approach: complete diffraction sets are assembled from a large number of individual diffraction frames acquired from small single unoriented crystals that are not cryoprotected (Guha *et al.*, 2012 ▶; Stellato *et al.*, 2014 ▶; Chapman *et al.*, 2011 ▶; Boutet *et al.*, 2012 ▶). Complete coverage of the Ewald sphere is obtained by assembling individual diffraction frames into a single data set. The ideal crystals for serial crystallography are large enough and sufficiently defect free to diffract to high resolution, are produced in large quantity, and are sufficiently identical to facilitate merging of diffraction frames.

Serial crystallography with non-cryocooled crystals has several technical advantages over conventional methods. First, the crystals can be small, which increases the potential for growing crystals in the first place. Second, it avoids the roughly tenfold increase in crystal mosaicity typically encountered during cryoprotection (Malkin & Thorne, 2004 ▶; Guha *et al.*, 2012 ▶) and eliminates the need to search for cryoprotectant conditions. Although non-cryoprotected crystals suffer radiation damage at roughly a hundred times higher rate than cryoprotected crystals (Garman, 2010 ▶), there is little dis­advantage associated with using many non-cryocooled crystals to obtain a complete data set if the crystals are easy to produce, plentiful and easy to handle.

**Figure 1:**
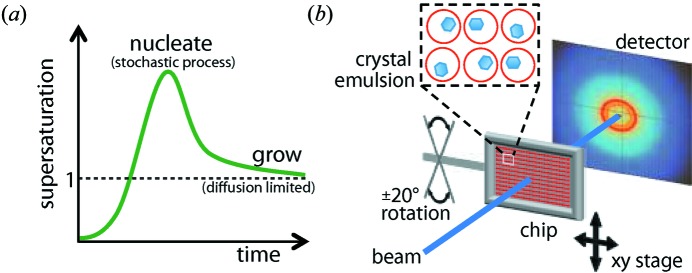
(*a*) An optimal crystallization trajectory increases supersaturation until just one crystal nucleates, then decreases supersaturation to prevent further nucleation while maintaining sufficient supersaturation to promote crystal growth. (*b*) Emulsion droplets with monodisperse crystals were stored in an X-ray semi-transparent microfluidic device. Sequentially collected diffraction frames from multiple individual crystals were merged to solve the protein structure. The chip could be translated in the *x* and *y* directions and rotated 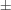
20°.

The ideal crystallization procedure, illustrated in Fig. 1 ▶(*a*), to produce protein crystals for any form of crystallography, including serial crystallography, consists of slowly increasing the supersaturation of a protein solution until the moment that a single crystal is nucleated. Then, once the first nucleation event occurs, the supersaturation is reduced enough to prevent further nucleation while maintaining sufficient supersaturation to grow the crystal. Ideally, the growth conditions should be slow enough to allow for annealing of defects, and the procedure must be capable of producing crystals in large numbers and of identical size. Additionally, the technology to produce crystals must be simple and inexpensive if serial crystallography is to be adopted by the structural biology community.

The challenge is to design such a method. The well known counter-diffusion method (Garcia-Ruiz, 2003*a* ▶,*b* ▶; Otalora *et al.*, 2009 ▶) produces a series of kinetic supersaturation profiles that rise and fall as illustrated in Fig. 1 ▶(*a*). However, both the time at which the supersaturation maximum occurs and the value of the supersaturation maximum are independent of the nucleation event. The maximum supersaturation varies along the capillary length, and with a long capillary the chances are improved that somewhere along the capillary there will be a location where the maximum supersaturation will coincide with the first nucleation event. However, this method requires long capillaries and is not optimal for volumes under 1 nl. Furthermore, counter-diffusion requires that the precipitant and protein have greatly different diffusion constants, so it is suitable for low molecular weight precipitants, such as salt, but not for macromolecules, such as poly(ethylene glycol) (PEG).

Another issue complicating design of the ideal profile of Fig. 1 ▶(*a*) is that at constant supersaturation nucleation is a random process, rendering it impossible to know *a priori* when to decrease supersaturation, which should coincide with the first nucleation event. One way to generate the ideal supersaturation profile would be to monitor the supersaturated solution with a technique, such as second-harmonic generation (SHG) microscopy (Wampler *et al.*, 2008 ▶; Kissick *et al.*, 2010 ▶), that is sensitive to the formation of small crystals and then, once the first crystal is detected, lower the supersaturation. However, this scheme will be cumbersome to implement in the high-throughput case of processing hundreds to thousands of samples. An alternative method is desired.

Microfluidically produced monodisperse emulsions have previously been used to produce drops of supersaturated protein solution in which each drop nucleates a single crystal (Zheng *et al.*, 2003 ▶; Ildefonso *et al.*, 2013 ▶; Selimović *et al.*, 2010 ▶; Dombrowski *et al.*, 2010 ▶). This situation is ideal for serial crystallography for a number of reasons. Since only one crystal nucleates per drop, all the supersaturated protein in solution is delivered to a single crystal, making that crystal as large as possible. Microfluidic precision allows preparation of emulsion droplets with variations in size of a few percent only, even at high flow rates (Romanowsky *et al.*, 2012 ▶). Furthermore, because of the small length scales in microfluidics, convection is suppressed and flows are laminar. Taken together, these factors mean that processing proteins using microfluidics leads to crystals of a uniform size that are grown under identical conditions, which has the effect of creating crystals that have similar characteristics, such as unit cell and degree of disorder. Having identical crystals facilitates merging of diffraction data sets taken from different crystals.

In the microfluidic device described here, we first produce drops containing protein. Then, exploiting surface tension forces, we guide the drops to 8000 storage sites on chip (Shim *et al.*, 2007 ▶; Schmitz *et al.*, 2009 ▶). Next, we increase supersaturation to induce crystallization in such a way as to produce one crystal per drop. Finally, we sequentially collect diffraction data from individual crystals and merge the data sets in order to solve the protein structure (Fig. 1 ▶ *b*).

Producing and diffracting from crystals in the same device eliminates the laborious and potentially damaging steps of looping and extracting the crystal from the mother liquor. Various microfluidic crystallization platforms compatible with *in situ* diffraction have been developed (Hansen *et al.*, 2006 ▶; Li *et al.*, 2006 ▶; Dhouib *et al.*, 2009 ▶; Guha *et al.*, 2012 ▶). However, these devices incorporated valves in the chip (Hansen *et al.*, 2006 ▶; Guha *et al.*, 2012 ▶), thus rendering them expensive to manufacture and difficult to operate. Other technologies are low throughput (Dhouib *et al.*, 2009 ▶) or need a second round of scale-up to larger capillaries (Li *et al.*, 2006 ▶) to produce crystals large enough to collect diffraction data.

## 2. One crystal per drop through compartmentalization   

The production of one crystal per drop is a result of a competition between two processes, nucleation and growth, in a confined volume. Both processes require supersaturation and therefore both nucleation and growth are nonequilibrium processes. When the first nucleus forms inside the drop, it decreases the supersaturation in the surrounding protein solution as the crystal grows. If the rate of nucleation is low enough, then the growing crystal will consume enough of the protein in solution to decrease the supersaturation to the point where another nucleation event is improbable. Further nucleation is prevented if the time for a protein to diffuse across a drop is less than the time to nucleate a crystal (Dombrowski *et al.*, 2010 ▶). Thus, combining a small drop volume with the physics of nucleation and growth generates negative feedback, which acts to create autonomously the ideal dynamic supersaturation profile that produces one crystal per drop. Instead of having the negative feedback imposed externally, as in the cumbersome SHG microscopy scheme discussed previously, here the negative feedback is engineered into each drop; no external intervention is required. All the engineering goes into identifying the correct combination of diffusive flux, nucleation rate and drop volume for the emulsions. A theoretical argument and computer simulations describing the processes leading to one crystal per drop in small volumes are detailed in Appendix *A* [App appa].

## 3. Crystal emulsions   

**Figure 2:**
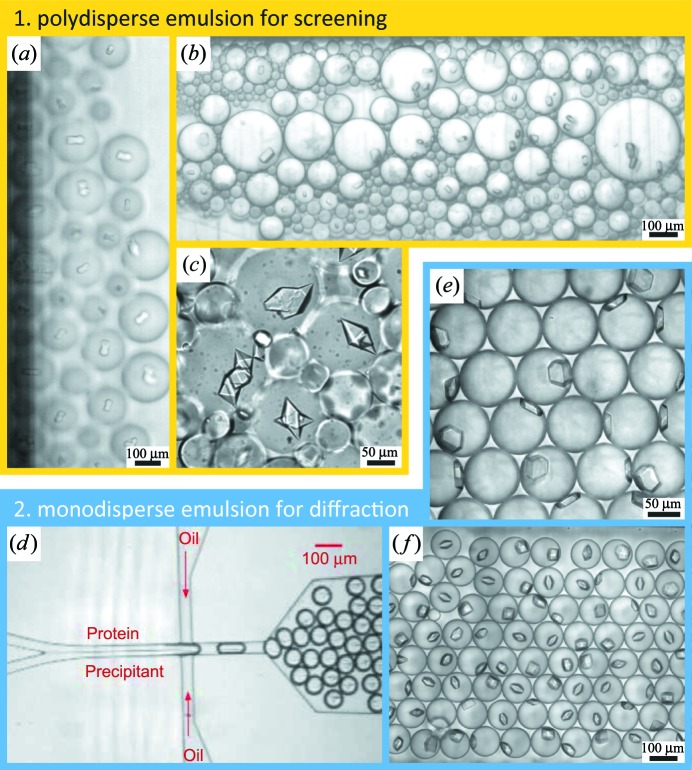
Protein crystallization in emulsion droplets stabilized by surfactant. Ideal drop sizes were first identified using polydisperse emulsion droplets. Monodisperse emulsions were used to produce identical crystals for diffraction experiments. Droplets were stored in a rectangular glass capillary. (*a*)–(*c*) Polydisperse emulsions of (*a*) D1D2 heterodimer from human spliceosomal snRNP particle, (*b*) concanavelin A and (*c*) trypsin. (*d*) Protein and precipitant solutions were introduced in a co-flow geometry under laminar flow conditions that prevent mixing upstream of the nozzle where both solutions became encapsulated into emulsion droplets. (*e*), (*f*) Monodisperse emulsions of (*e*) glucose isomerase and (*f*) lysozyme crystals. See main text for crystallization conditions. (*d*) and (*f*) are from Akella (2014 ▶).

To yield identical crystals in sufficient quality and quantity for serial crystallography, we use a two-step method. We first identify the appropriate drop volume to nucleate one crystal per drop consistently. For this we intentionally created emulsions in a batch process that yielded a polydisperse size distribution, ranging from a few micrometres to a few hundreds of micrometres in diameter (Figs. 2 ▶ *a*–2 ▶ *c*). Such a polydisperse emulsion allowed us to identify the appropriate drop diameter in a single screening experiment. We then used microfluidics (Fig. 2 ▶ *d*) to produce monodisperse emulsion droplets (Figs. 2 ▶ *e* and 2 ▶ *f*), which we used to grow identical crystals in the serial X-ray diffraction chip, as described in §[Sec sec5]5. For the purposes of this paper, however, the full experimental sequence will only be reported for glucose isomerase, *i.e.* whereby crystals were grown in the serial diffraction chip, X-ray data were acquired and the structure was solved.

All crystals were grown in emulsion droplets stabilized against coalescence with a 2%(*v*/*v*) solution of PFPE–PEG–PFPE surfactant ‘E2K0660’ (PFPE is perfluoropolyether) in HFE7500 fluorinated oil (from 3M). The surfactant was synthesized as previously described (Holtze *et al.*, 2008 ▶). Note that a commercial surfactant, which we have used in other experiments, is now available (RAN Biotechnologies; http://www.ranbiotechnologies.com). We chose a fluorinated oil and surfactant to minimize interactions with biological molecules. Fluorocarbon and hydrocarbon oils do not mix with each other, nor do they mix with water. In particular, the PFPE–PEG–PFPE surfactant in HFE7500 oil system has been shown to have excellent biocompatibility (Holtze *et al.*, 2008 ▶; Sanchez *et al.*, 2012 ▶). To confirm that it is compatible with protein crystallization, we tested it with five crystallization model proteins (Fig. 2 ▶ and Table 1 ▶). All five model proteins have previously been crystallized by vapor diffusion and a structure derived from X-ray crystallography deposited in the Protein Data Bank (PDB; Berman *et al.*, 2000 ▶).

To adopt a published vapor diffusion recipe into our emulsion format we had to perform a set of pre-experiments. In traditional vapor diffusion, a small volume of protein solution is mixed with the same amount of precipitant and then sealed into a container together with a large reservoir of precipitant. The diluted protein–precipitant drop equilibrates through vapor phase diffusion with the reservoir, resulting in a concentration increase of all components in the drop by approximately a factor of two. All previously published crystallization recipes had been optimized to nucleate only a few crystals per microlitre. Our emulsion droplets have volumes of a few pico- to nanolitres each. As the probability of nucleating a crystal is proportional to the sample volume, we had to increase nucleation rates by at least two orders of magnitude. We thus prepared vapor phase and microbatch crystallization trials around the literature recipes and optimized the vapor recipes toward nucleating crystal showers of appropriate density. When attempting to crystallize a novel protein target through screening crystallization conditions, such crystal showers are usually considered a first hit and the conditions are later refined extensively to grow the largest possible crystal. When using the method presented here on a novel protein target, the polydisperse emulsion screen can be used directly with conditions giving crystallites and crystal showers commonly identified as first hits in vapor diffusion screens. This would eliminate the reverse engineering step of converting an optimized vapor phase recipe back to a recipe that grows crystal showers.

Polydisperse emulsions were then prepared by mixing 2 µl of protein solution with 2 µl of precipitant in a 150 µl PCR test tube. Immediately after mixing, we added 30 µl of 2%(*v*/*v*) solution of PFPE–PEG–PFPE surfactant (E2K0660) in HFE7500 fluorinated oil. Polydisperse emulsions were formed by gently agitating the vial by hand until droplets became too small to be resolved by eye. This procedure typically gave droplets ranging from a few micrometres to a few hundreds of micrometres in diameter (Figs. 2 ▶ *a*–2 ▶ *c*). The aqueous droplets were less dense than the immersing fluorinated oil, so the droplets rose (‘creamed’) to the top of the vial within a minute. The emulsion cream was then loaded into rectangular glass capillaries (VitroTubes from VitroCom, Mountain Lakes, NJ, USA) and sealed with 5 Minute Epoxy to prevent evaporation. Crystallization was monitored over the course of a week. All compounds and proteins from commercial sources were used as received without further purification. The molecular weight and the net charge of the proteins during crystallization, as derived from the isoelectric point, are summarized in Table 1 ▶. To first order, preparing a polydisperse emulsion takes about the same time as preparing a hanging or sitting drop vapor diffusion condition. Both require three pipetting steps and a final lidding or shaking operation. We also successfully used conventional pipetting robots and 96-well plates for emulsion screening.

Lysozyme was crystallized by encapsulating 30 mg ml^−1^ lysozyme, 100 m*M* sodium acetate pH 4.8, 12.5 wt% PEG 8000, 5 wt% NaCl (all Sigma Aldrich) final concentration into droplets and then incubating them at 279 K for 36 h until all droplets had nucleated crystals (Akella, 2014 ▶). This recipe was derived from a vapor phase recipe mixing 20 mg ml^−1^ lysozyme in 100 m*M* sodium acetate pH 4.8 with an equal volume of 10%(*w*/*v*) NaCl, 100 m*M* sodium acetate pH 4.8, 25%(*v*/*v*) ethylene glycol (Rigaku, 2013 ▶).

Glucose isomerase crystals were grown at room temperature (∼298 K) within two days by preparing a crystallization batch with final concentrations of 30 mg ml^−1^ glucose isomerase from *Streptomyces rubiginosus* (from Hampton Research), 100 m*M* ammonium sulfate pH 7.0, 20 wt% PEG 10 000 in a 1:1 ratio (all from Sigma Aldrich). The initial vapor phase crystallization condition was taken from the Hampton Research data sheet as mixing 20–30 mg ml^−1^ glucose isomerase with 10–15%(*w*/*v*) PEG 4000–8000, 200 m*M* salt pH 6.0–9.0.

Trypsin was crystallized by mixing 60 mg ml^−1^ trypsin (Sigma T-8253) from bovine pancreas in 10 mg ml^−1^ benzamidine, 3 m*M* CaCl_2_, 0.02 wt% sodium azide with 100 m*M* NaPO_4_ pH 5.9, 5.1 *M* ammonium acetate (all Sigma Aldrich). In this system we observed crystals within one day in the range of pH 5.9 to pH 8.6, with higher pH values having much higher nucleation rates. This recipe was derived from a vapor phase recipe mixing 60 mg ml^−1^ trypsin in 10 mg ml^−1^ benzamidine, 3 m*M* calcium chloride, 0.02%(*w*/*v*) sodium azide with an equal volume of 4%(*w*/*v*) PEG 4000, 200 m*M* lithium sulfate, 100 m*M* 2-(*N*-morpholino)ethanesulfonic acid pH 6.5, 15% ethylene glycol (Rigaku, 2013 ▶).

Concanavalin A was crystallized by mixing 25 mg ml^−1^ concanavalin A type IV from *Canavalia ensiformis* in 10 m*M* Tris hydrochloride pH 7.4 with 100 m*M* Tris hydrochloride pH 8.5, 8 wt% PEG 8000 in a 1:1 ratio (all from Sigma Aldrich). For this we first set up vapor phase and microbatch trials of 20 mg ml^−1^ concanavalin A in 10 m*M* Tris pH 7.4 against the 50 conditions in the Hampton Crystal Screening Kit. From this screen we choose condition 36, with 100 m*M* Tris hydrochloride pH 8.5, 8%(*w*/*v*) PEG 8000, as this condition grew crystals in both vapor phase and microbatch trials.

D1D2, the sub-complex from the human snRNP spliceosome core particle (PDB entry 1b34; Kambach *et al.*, 1999 ▶), crystallized over a period of 72 h at room temperature from a crystallization batch with final concentrations of 6 mg ml^−1^ D1D2, 62 m*M* sodium citrate pH 5.2, 125 m*M* ammonium acetate, 9 vol.% glycerol, 26%(*w*/*v*) PEG 4000 (all Sigma Aldrich). D1D2 was purified as previously reported (Kambach *et al.*, 1999 ▶). D1D2 was first crystallized by Kambach *et al.* (1999 ▶) in vapor phase by mixing equal volumes of 6 mg ml^−1^ D1D2 in 20 m*M* sodium HEPES pH 7.5, 200 m*M* sodium chloride and 6 m*M* dithiothreitol and 100 m*M* sodium citrate pH 5.6, 200 m*M* ammonium acetate, 15% glycerol, 25% PEG 4000.

All globular proteins, concanavalin, glucose isomerase and trypsin, crystallized readily in vapor diffusion, microbatch and the emulsion system. The heterodimer D1D2 formed crystals in the vapor phase and the emulsion system only. In microbatch a thick protein skin grew at the droplet interphase, potentially depleting all the protein from the drop. We thus conclude that the PFPE–PEG–PFPE surfactant system is well suited to protecting protein from adsorbing at the fluoro oil–water interface and to stabilizing emulsions, making it ideal for crystallization trials. Future work should investigate the compatibility of the surfactant with other proteins. In particular, in membrane protein crystallization, the crystallization cocktail includes surfactants, which may partition to the fluoro oil–water interface and affect either the protein or emulsion stability. However, the two membrane proteins porin from *Rhodobacter capsulatus* and reaction center from *Rhodo­pseudomonas viridis* have previously been crystallized in a related fluorinated FC-40 and FC-70 oil (Li *et al.*, 2006 ▶).

All initial crystallization experiments were performed at room temperature. However, a particular protein may become unstable at too high or too low temperatures. Also, many proteins like lysozyme have temperature-sensitive nucleation rates, which one might like to exploit in crystallization trials (Akella, 2014 ▶). An ideal surfactant–oil system can hence be used in a large temperature range. To test for temperature compatibility, we prepared crystal emulsions from the PFPE–PEG–PFPE surfactant in HFE7500 oil, sealed them into rectangular glass capillaries, and incubated them in a water bath at 277 K and at 313 K. We found the emulsion droplets to be stable for at least two weeks at those two temperatures.

Finally, to yield identical crystals in sufficient quantity for serial crystallography, we employed microfluidics to produce monodisperse emulsion droplets. For this we simply selected the drop-making chip appropriate to make drops of the desired diameter and used the crystallization recipe from the preceding polydisperse emulsion screen without further modification. We produced drops in a co-flow geometry designed such that the protein solution and buffer do not mix in the laminar flow upstream of the dropmaker (Fig. 2 ▶ *d*). Typically, injection of the oil–surfactant mixture proceeded at 600 µl h^−1^, while both protein and precipitant streams were pumped at equal flow rates of 300 µl h^−1^ to co-encapsulate both in a 1:1 mixture. Upon droplet formation, mixing inside each droplet proceeds within less than a second owing to recirculating flow that arises from shearing interactions of the fluid inside the drops with the stationary wall (Tice *et al.*, 2003 ▶). These monodisperse emulsion droplets were then injected into and incubated in the diffraction chip to grow crystals for the X-ray diffraction experiments.

To monitor crystallization, we stored emulsion droplets in two different systems. Firstly, polydisperse emulsions were usually sealed into rectangular glass capillaries, which prevented water and oil evaporation. Secondly, as our diffraction chip was made from a polymer material, we exploited its permeability to water vapor by slowly letting droplets shrink by permeation of water from the drops into the oil and also from the drops through the thin polymer-based chip. Water permeation across the polymer foil is proportional to the permeation constant of the material and inversely proportional to the foil thickness (Shim *et al.*, 2007 ▶). In the case of the 25–75 µm-thick cyclic olefine copolymer (COC) sheets used here, the evaporative water loss amounted to a few percent per hour. When water evaporates from the drop, the solute concentrations inside the drop increase and hence the protein supersaturation also increases. As this corresponds to an increased nucleation rate, one would expect to yield a larger fraction of droplets with multiple crystals. We did not observe such an effect and attribute this to the fact that, once the first crystal nucleates, its subsequent growth reduces the supersaturation of the solution enough to prevent another crystal from nucleating. We consistently achieved one crystal per drop, which argues for the robustness of the method. Once all droplets had nucleated crystals after a few hours or days, we immersed the capillary/chip into an oil bath to prevent further evaporation (Li *et al.*, 2006 ▶). Alternatively, we achieved equally good results with storing chips in a water bath, while having a vial filled with oil connected to the chip and all other inlets sealed.

## 4. X-ray semi-transparent chip fabrication   

**Figure 3:**
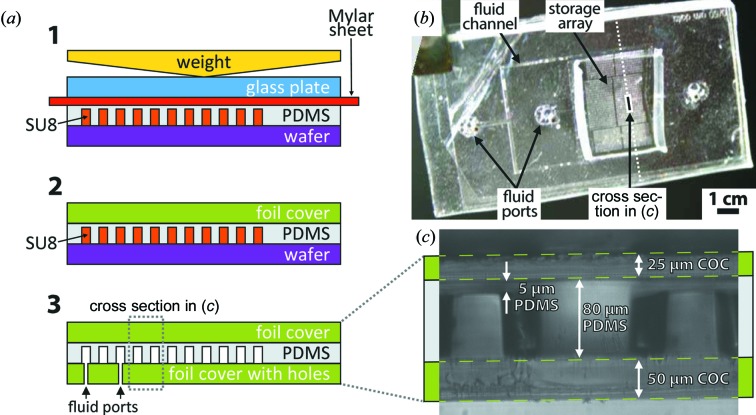
Chip fabrication. (*a*) PDMS resin was squeezed into a thin layer onto the SU8 master (1). After curing, a foil cover was bonded onto the featured PDMS using a silane coupling chemistry (Tang & Lee, 2010 ▶) (2). Then the reinforced PDMS film was peeled off and the chip was lidded using another foil cover (3). (*b*) Top view and (*c*) cross section of a device made from COC foil and PDMS. The cross section in (*c*) was obtained by cutting the chip across the storage array into two and was imaged by placing the chip edge on onto the microscope stage to magnify the cut. The chip shown here had a 5 mm-thick PDMS frame manifold for fluid interfacing where tubing could be directly inserted into the through holes in the PDMS.

A detailed description of the fabrication of the microfluidic chips is given by Guha *et al.* (2012 ▶). An overview and modifications for *in situ* X-ray diffraction follow. Chips were sealed, which is colloquially referred to in the thermoplastic industry as ‘lidding’, by bonding COC or Kapton foil to both sides of the thin poly(dimethylsiloxane) (PDMS) slab containing the channels (Fig. 3 ▶). PDMS (Sylgard 184 from Dow Corning) with a 1:5 ratio of curing agent to base was molded on a standard SU8 master (McDonald *et al.*, 2000 ▶) by squeezing the uncured PDMS resin into a thin film using a glass plate and a weight. To facilitate release of the PDMS film, the master was surface-treated with a fluorophilic coating by spin coating 1:20 Cytop CTL-809M in CTsolv.100E (both Bellex International) onto the master. We then baked the wafer for 1 h at 423 K. We placed a 30 µm-thick Mylar foil (DuPont) between the PDMS and the glass to allow for easy removal of the glass slide after PDMS curing. We pre-cured the PDMS for 4 h at room temperature before we removed the weight and transferred the complete stack into an oven to drive the curing reaction to completion at 345 K for another hour.

We used either COC (TOPAS 5013 from Advanced Polymers) or Kapton (American Durafilm), depending on experimental requirements. COC is more brittle then Kapton but has a lower water vapor permeability. The thinnest commercial COC we used was 25 µm-thin TOPAS, while Kapton as thin as 8 µm can be purchased as bulk foil. We chemically bonded either substrate to the featured PDMS using a silane coupling chemistry (Tang & Lee, 2010 ▶). In brief, the foil and PDMS were both activated in an oxygen plasma and then each incubated separately for 25 min in an aqueous solution of a different silane: one in 1 vol.% of 3-amino­propyl­tri­methoxy­silane (APS; 97% from Aldrich) and the other in 1 vol.% of 3-glycidoxy­propyl­tri­methoxy­silane (GPS; 98%, from Aldrich). The two silanes are such that they can form an epoxy bond when brought into contact. The method works equally well with the foil treated with APS and the PMDS with GPS, or *vice versa*. Upon removing the foil and PDMS from the batch, we dried both with a stream of nitrogen gas and then carefully brought them into contact using tweezers to prevent trapping air bubbles between the two layers. The chip was then incubated in the oven at 345 K for 1 h to maximize chemical cross-linking. The process was repeated to lid the other side of the chip, now with a foil that had through holes at the appropriate locations for fluid inter­facing. Through holes were punched using a 0.75 mm Harrison Uni-Core biopsy punch (Ted Pella). Upon assembly the chip was surface-treated with a fluoro­philic coating to prevent protein interaction with the channel surface. For this, 1:20 Cytop CTX-109AE in CTsolv.100E (both Bellex International) was dead-end filled into the chip by plugging all outlets and slowly injecting the Cytop solution through the inlet into the chip. This causes gas bubbles trapped inside the chip to become pressurized, which prompts the gas to dissolve into the solution and also to permeate across the chip walls to result in a completely filled bubble-free device. The chip was then incubated at 363 K for at least 12 h to evaporate the solvent away and also to accelerate chemical cross-linking between the fluoropolymer and the chip surface.

## 5. *In situ* diffraction   

**Figure 4:**
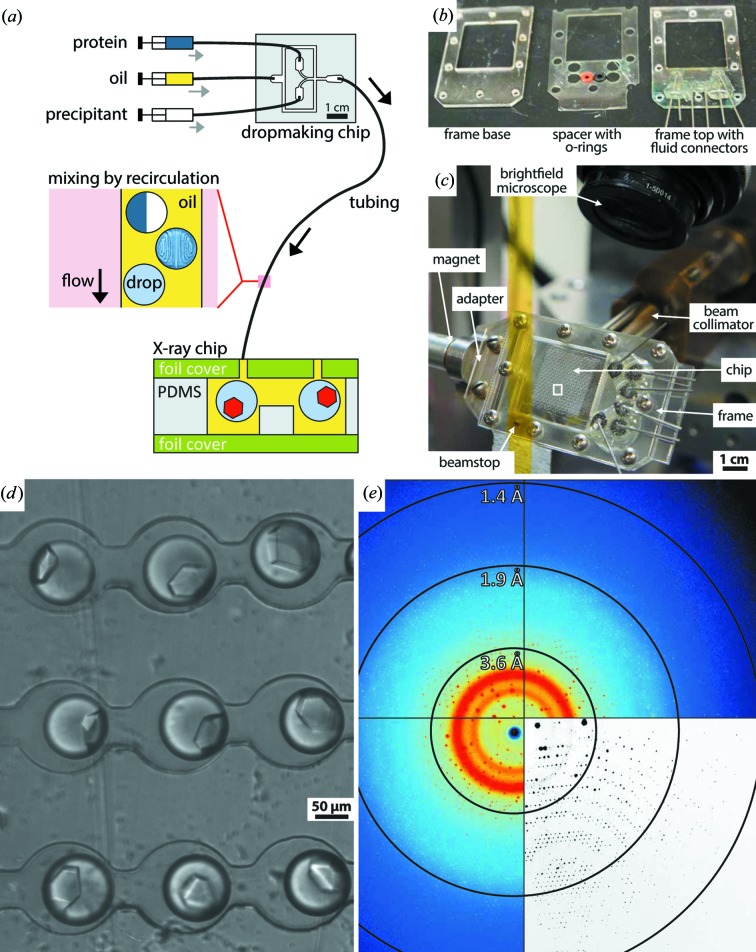
(*a*) Monodisperse emulsions were prepared using a dedicated dropmaking chip as illustrated in Fig. 2 ▶(*d*) and directly routed into the chip for serial crystallography for storage. (*b*) We used a laser-cut frame to hold and to port into the X-ray semi-transparent chip. (*c*) The X-ray semi-transparent chip mounted on the goniometer inside the Cornell CHESS F1 beamline. (*d*) Glucose isomerase crystals inside of the microfluidic device. Using a motorized stage, each crystal can be centered in the collimated X-ray beam. The beam is 100 µm in diameter. (*e*) A representative diffraction pattern of a glucose isomerase crystal taken at room temperature from inside the chip. Crystals diffracted to 1.4 Å resolution with a mosaicity as low as 0.04°. The bottom-right quadrant shows the diffraction pattern after background subtraction, using the *Adxv* diffraction pattern visualization tool (http://www.scripps.edu/~arvai/adxv.html) with subtract background option.

We mounted the X-ray-transparent chip into a custom acrylic frame to collect diffraction data (Fig. 4 ▶). The acrylic frame was cut to shape from 3 mm-thick acrylic sheet using a 40 W CO_2_ Hobby Laser cutter with a 1.5′′ (1′′ = 25.4 mm) focus lens (Full Spectrum Laser). To create ports into the foil chip we drilled through holes into the acrylic frame with the laser cutter. Blunt needle tips (23 gauge) were then placed into the holes and glued into position with 5 Minute Epoxy. We connected #30 AWG poly(tetrafluoroethylene) tubing (Cole Palmer) to the needle tips using PDMS cubes with through holes punched into them by a 0.75 mm Harrison Uni-Core biopsy punch (Ted Pella). Buna O-rings, 70 durometer, size 002 (McMaster Carr), were then used to seal the foil chip to the hollow metal pins. For easy alignment the O-rings were fitted into a 1 mm-thick poly(ethyleneterephthalate) spacer that also was fabricated with the laser cutter. X-ray semi-transparent foil chips were mounted into a frame for the duration of each experiment. Each frame was held together by ten self-tapping 3/16′′ Pan Head 2-28 Phillips screws (McMaster Carr) to lock the chip into position and to minimize flow induced inside the chip from mechanical bending of the thin-foil chip. To mount the frame–chip assembly in the synchrotron we machined a stainless steel adapter that a frame could be mounted onto using two screws (Fig. 4 ▶ *b*).

For the proof-of-principle experiment we fabricated an X-ray semi-transparent chip with the ‘dropspot’ geometry (Schmitz *et al.*, 2009 ▶) that can hold up to 8000 emulsion droplets in cavities with 150 µm diameter each (Figs. 3 ▶ *b* and 3 ▶ *c*). The fluorinated oil has a density of 2 g ml^−1^, while the water drops have a density of 1 g ml^−1^. Thus there is a strong tendency for the drops to float to the top of the oil, or ‘cream’. Surface tension forces arrest droplets in a cavity and prevent them from creaming to one side of the chip. We produced a monodisperse ∼110 µm-diameter emulsion of 30 mg ml^−1^ glucose isomerase, 100 m*M* ammonium sulfate pH 7.3, 20 wt% PEG 10 000 *M*_W_ final concentration in a standard dropmaker (Fig. 2 ▶ *d*). Droplets exiting the dropmaker were immediately routed into the X-ray semi-transparent serial crystallography chip by simply plumbing the dropmaker outlet into the dropspot inlet (Fig. 4 ▶ *a*). After the dropspot chip was loaded, we dead-end plugged its outlet except for one inlet where we kept HFE7500 oil entering the chip using hydrostatic pressure to compensate for oil evaporation from the chip. We incubated the chip at room temperature for three days and monitored crystallization, before transferring into a water bath to prevent further evaporation. By then, most droplets had shrunk to about ∼90 µm diameter and more than 90% of them had nucleated a single crystal. Crystals grew to about 50 × 40 × 30 µm in size at room temperature (∼298 K).

X-ray diffraction data were collected at Cornell High Energy Synchrotron Source, beamline F1 (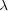 = 0.9179 Å, 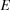 = 13.508 keV, X-ray flux = 5.53 × 10^10^ photons s^−1^), using a 100 µm monochromatic X-ray beam from a 24-pole wiggler. The chips were mounted at a distance of 200 mm from an Area Detector Systems Corporation Quantum 270 detector, corresponding to a largest inscribed circle of resolution of 1.4 Å. The detector face was oriented perpendicular to the beam. For selected crystals within the chip, data sets were collected at room temperature (∼295 K). Each recorded data set comprised ten frames, for a total of 10° oscillation. Each image consisted of a 5 s exposure with a 1° oscillation step size. A total of 1520 images were collected from 152 glucose isomerase crystals in three different dropspot chips.

## 6. X-ray structure determination   

The software *HKL-2000* was used to index, refine, integrate and scale each 10° data set (Otwinowski & Minor, 1997 ▶) before merging. Parameters including unit-cell size, χ^2^ values, resolution, mosaicity and completeness were evaluated for every partial data set during the indexing and scaling process. From these partial data sets, with 1520 frames total, we selected 262 frames from 72 crystals by rejecting frames with a mosaic spread higher than 0.1° and χ^2^ 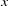 and 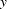 (corresponding to the discrepancy between observed and predicted spot positions) above 2. Some frames were later rejected because of poor scaling statistics; the final data set included 248 frames.

Glucose isomerase crystals were determined to have a space group of *I*222 and diffracted to an average of 2 Å; an example image is shown in Fig. 4 ▶(*e*). In some crystals, diffraction extended to 1.4 Å, with a mosaic spread of 0.04.

The 248 selected frames were scaled together using *SCALEPACK* (HKL Research, Charlottesville, VA, USA) and merged with *Aimless* (Evans, 2011 ▶). The limiting resolution of 2.09 Å was chosen as that at which CC1/2 dropped below 0.5. Statistics are given in Table 2 ▶. The merged data set covered 93% of reciprocal space, suggesting that preferred orientation of the crystals was not a major problem. The glucose isomerase structure was readily solved by molecular replacement with *MOLREP* (Vagin & Teplyakov, 1997 ▶) using the structure previously determined at 1.90 Å resolution (PDB entry 8xia; Carrel *et al.*, 1989 ▶), with water molecules removed. Prior to refinement, we randomly flagged 5% of the reflections for 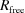 analysis (Brunger, 1992 ▶).

**Figure 5:**
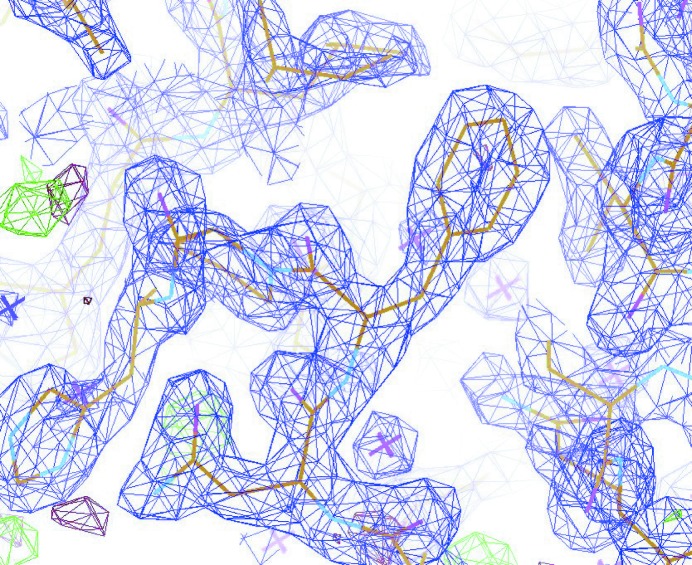
Part of the final refined structure showing the quality of the electron density map. The 2*F*_o_–*F*_c_ map is shown in purple, contoured at 2σ, while the *F*_o_–*F*_c_ map is shown in red (negative) and green (positive), contoured at 3σ.

Structure refinement was carried out through multiple iterations of *REFMAC* (Murshudov *et al.*, 2011 ▶), refining atomic coordinates and isotropic *B* factors. 2*F*_o_–*F*_c_ and *F*_o_–*F*_c_ electron density maps were generated after each refinement step, and further refinement was carried out by manual inspection using *Coot* (Emsley & Cowtan, 2004 ▶). In the refinement process, two disordered *N*-terminal residues were removed, as well as a bound sugar molecule present in the model but not in the crystal, and 124 water molecules were added. Final refinement gave *R* and *R*_free_ values of 0.144 and 0.174, respectively. Complete processing statistics are given in Table 3 ▶. Fig. 5 ▶ shows the quality of the final refined structure.

## 7. Conclusion   

Here, we present a technology that optimizes the kinetics of crystallization, eliminates crystal handling, eliminates cryoprotection and simplifies collection of diffraction data for structural biology. In this paper we developed processing methods for protein crystallization that follow the ideal kinetic pathway of slowly increasing supersaturation until a single crystal nucleates and then reducing supersaturation so that one crystal grows slowly to allow annealing of defects. Sample volume is not a thermodynamic variable in phase equilibrium, but since crystallization is a non-equilibrium process, volume plays a key role in determining the kinetics of crystallization. In Appendix *A* [App appa], we argue using a combination of simulation, theory and experiment that selecting the appropriate droplet diameter guarantees that only one crystal per drop will form when the drop volume 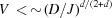. We identify the critical drop diameter for a particular crystallization condition in a single experiment by using a polydisperse emulsion with droplets ranging from a few micrometres to a few hundreds of micrometres in size. These polydisperse emulsions can be made with ease within seconds using only a pipette and a test tube. The probability of crystallization is proportional to the drop volume. As we use drops of order 1 nl, which are smaller drops than employed by other methods, the nucleation rates and supersaturation that we use are higher than usual. It remains to be seen how such high nucleation and growth rates impact crystal quality. In the future we will study the quality of protein crystal structure determination as a function of crystal size, nucleation rate and crystal growth rate to determine the optimal crystal size and crystallization conditions for serial crystallography.

Employing these kinetic processing methods, we grew monodisperse crystals compartmentalized in emulsion droplets, with one crystal per drop. Monodisperse microfluidically produced drops of supersaturated protein solutions were stored on chip and slowly concentrated as water permeated through the thin-foil chip. One single crystal per drop was nucleated and grown on chip in identical conditions. While cyrocooled crystals can be stored almost indefinitely, the crystals grown and stored in our chips are stable for several weeks when the chips are stored in a water bath connected to an oil reservoir, which prevents evaporation and hence drying out. The chip for nucleating crystals was thin enough to be X-ray semi-transparent, and diffraction patterns were collected from these crystals on chip at room temperature. The structure of glucose isomerase was solved and refined at 2.09 Å resolution, to an 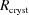/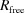 of 0.144/0.174, using merged diffraction data sets from 72 crystals of about 50 × 40 × 30 µm in size.

Diffraction from room-temperature crystals stored on the chip in which they were nucleated and grown has many advantages over traditional off-chip cyroprotected crystals. On-chip diffraction means the crystals are not removed from their mother liquor, thus avoiding a process that can lead to dehydration and osmotic shock of the crystals and the generation of stress and strain. Room-temperature diffraction eliminates the laborious step of cryoprotection and has the additional effect of lowering the mosaicity, as cryoprotection generates stresses as a result of changing solvent conditions and temperature-induced volume changes. Our chip can be inexpensively mass produced and is simple to operate without the need for controlling valves.

The long-term vision is to create a chip that uses temperature and concentration gradients to discover optimal crystal growth conditions (Shim *et al.*, 2007 ▶). Next, crystals would be grown at the optimal conditions to create a stream of tiny crystals that would be serially conveyed to a part of the chip with ultra-thin windows for *in situ* diffraction. For this we are exploring windows made from materials such as ultra-thin silicon nitride (Weinhausen & Köster, 2013 ▶) or graphene (Wierman *et al.*, 2013 ▶) and ways to reduce in-beam volumes of the fluids surrounding crystals.

## Figures and Tables

**Figure 6:**
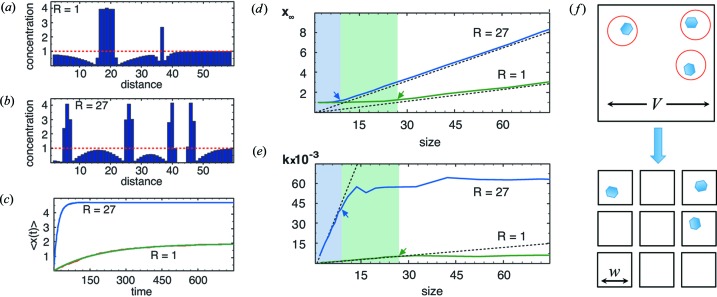
(*a*), (*b*) Protein concentration as a function of distance from a simulation of nucleation and growth in one dimension. The concentration is dimensionless. The red dotted line indicates the initial concentration with a supersaturation of 83.3 at 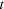 = 0. The sites with concentrations that exceed the red line are in the crystalline phase, while those below are in solution. (*a*) Concentration profile at 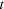 = 250. Slow nucleation rate of 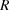 = 1 in dimensionless units. (*b*) Concentration profile at 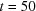. Fast nucleation rate of 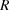 = 27. (*c*) The average number of crystals per drop as a function of time, 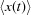, for two nucleation rates obtained from simulation and fitted to equation (3), 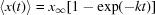. The conditions are the same as in (*a*) and (*b*). (*d*), (*e*) Fitting parameters to equation (3) as a function of drop size for two nucleation rates, 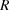 = 27 and 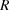 = 1. Arrows indicate the size of the depletion zone. (*d*) The solid lines are the simulated final number of crystals per drop, 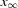. The dashed lines are equation (7), 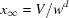. (*e*) The solid lines are the simulated rate of crystal formation, 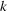. The dashed lines are equation (8), 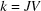. (*f*) Conceptual schematic. A drop of volume 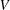 can be thought of as 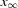 smaller independent drops of volume 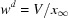

**Table 1:** Properties of crystallized proteins The theoretical pI value of D1D2 was computed using its amino acid sequence and the ExPASy *ProtParam* tool (http://web.expasy.org/protparam).

	Formula weight (kDa)	Isoelectric point (pI)	Net charge in crystal
Lysozyme	14.3	11.3 (from Wetter & Deutsch, 1951 ▶)	Positive
Trypsin	24	10.1–10.5 (from Walsh, 1970 ▶)	Positive
Concanavalin A	76.5 (3mer)	4.5–5.5 (multiple isoforms, see Entlicher *et al.*, 1971 ▶)	Negative
Glucose isomerase	173 (4mer)	3.95 (from Vuolanto *et al.*, 2003 ▶)	Negative
D1D2	26.8 (heterodimer)	10.6 (theoretical pI, from *ProtParam*)	Positive

**Table 2:** Processing results of merging the 248 frames obtained from 72 glucose isomerase crystals Values in parentheses refer to the highest resolution bin (2.15–2.09 Å).

Precipitant composition	100 m*M* ammonium sulfate pH 7.0 + 20 wt% PEG 10 000
Space group	*I*222
Unit-cell parameters (Å)	*a* = 93.94, *b* = 99.47, *c* = 102.85
Resolution range (Å)	49.7–2.09 (2.15–2.09)
No. of unique reflections	26 699 (2075)
Redundancy	8.2 (8.1)
Completeness (%)	93.2 (94)
*R*_merge_	0.191 (0.686)
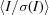	7.8 (4.1)
Mosaicity (°)	0.03–0.1

**Table 3:** Refinement and model statistics for glucose isomerase Values in parentheses refer to the highest resolution bin.

Resolution range (Å)	49.7–2.09 (2.14–2.09)
Reflections used: working, total	25 395, 26 685 (1879, 1974)
Completeness (%)	92.4 (93.6)
*R*(working)/ 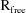	0.144/0.174 (0.186/0.227)
RMSD, bond lengths (Å)	0.019
RMSD, bond angles (°)	1.93
No. of protein/other atoms (non-H)	3034/126
Mean *B* value, all atoms (Å^2^	17.6
Ramachandran statistics (%): favored, allowed, outliers	97.13, 2.35, 0.52

*R* and 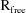 are calculated using 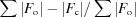 for the working and free-set reflections, respectively.

## Appendix A One crystal per drop: theory and simulation

In this section we calculate the drop volume such that only one crystal is nucleated per drop. Consider a drop that contains a supersaturated solution that has not nucleated any crystals. As long as the physical–chemical environment is constant, the nucleation rate,  (number of crystals per unit volume, , and per unit time, ), will also be constant and the probability, , of nucleating a crystal in a drop of volume  in an infinitesimal time interval  is independent of the time, :

from which it follows that the probability that a drop has not nucleated any crystals is . If, by some contrivance, each drop could only produce one crystal, then since the probability of not crystallizing and the probability of crystallizing have to add to one, we have an expression for the average number of crystals per drop as a function of time:

However, once a drop does nucleate a crystal, the nucleation rate is reduced because the growing crystal consumes protein in solution and nucleation ceases to be a Poisson process. This makes finding an analytical solution to the number of crystals per drop as a function of time a difficult problem (Dombrowski *et al.*, 2010 ▶; Goh *et al.*, 2010 ▶).

To address the question of how many crystals nucleate per drop as a function of drop size, we developed a Monte Carlo simulation in one dimension, a special case for which the drop size and volume are equal. Our approach differs from that taken previously by Dombrowski *et al.* (2010 ▶) in that our model explicitly calculates the spatial–temporal concentration profile within the drop. Drops were modeled as a lattice of points, where each point was characterized by two quantities: the protein number concentration,  (l), and a binary indicator that signified whether the protein was in a crystalline or solution state. The protein was confined in the drop, meaning that no-flux boundary conditions were imposed on the ends of the lattice. The numerical values used in the model, while within an order of magnitude of the values used in our experiments, were not reflective of any particular protein or physical set of conditions. Rather they were chosen for two purposes: first, to satisfy the assumptions of the theory, *i.e.* that the rate of crystal growth was much larger than the rate of nucleation; second, to ensure that the simulations were quick to perform. Thus the diffusion constants and nucleation rates were chosen to be higher than the actual values. This means that the simulations were faster to perform but that their conclusions were not affected, as the result depends on the ratio of diffusion rate to nucleation rate and not on their absolute values. Protein concentrations in solution evolved according to the diffusion equation , with *D* = 6 × 10^−10^ m^2^ s^−1^ the protein diffusion constant. Initially the drop was homogeneous in protein number concentration, *c* = 1 µm^−3^, at a high value of supersaturation, *s* = 83.3, with *s* = *c*/*c*_s_, where *c*_s_ = 0.012 µm^−3^ is the concentration of the saturated protein solution in equilibrium with the protein crystal. At each time step, there was a finite probability that a randomly chosen lattice site could transform into a crystal with a probability *P*, given by *P* = *Jl* ^3^τ, where  is the simulation time step and *l* = 1 µm is the size of a lattice site. In the simulation we used the classical nucleation theory expression for nucleation rate,  (Galkin & Vekilov, 1999 ▶), where *A* and *B* are constants such that , with *R* a dimensionless rate coefficient. The protein concentration of a lattice site co­inciding with the edge of a crystal was increased at a rate proportional to the supersaturation according to , where  m s^−1^ is the constant speed of crystal growth (Schmit & Dill, 2012 ▶; De Yoreo & Vekilov, 2003 ▶). Conservation of mass was used at the boundary between crystal and solution. The concentration per lattice site in a growing crystal was limited to an arbitrary value of  = 4 to model the effect that protein crystals have a fixed density that is of the order of 100. Once a lattice site exceeded this maximum concentration, the crystal would grow symmetrically, one lattice site to the right and one to the left. It was assumed that crystals were stationary once nucleated.

Figs. 6 ▶(*a*) and  6 ▶(*b*) show the simulation results. Parameters were chosen to approximate our experiments: high supersaturation, fast growth and drops of the order of 100 µm diameter. In each case, the simulation begins by instantly quenching the drop to a supersaturation of 83. The two figures correspond to a point after nucleation has occurred but before equilibrium is achieved. In Fig. 6 ▶(*a*) the nucleation rate is low,  in dimensionless units. In what follows, time, , is nondimensionalized by , and distance is non­dimensionalized by  = 1 µm. The red dashed line indicates the initial condition, at , while Fig. 6 ▶(*a*) shows the situation at  = 250. The crystal that nucleated first is centered at  = 19. The width of the crystal increases as protein from the solution is fed into the crystal. Later a second crystal is independently nucleated. The growing crystals deplete the protein concentration in the region bordering the crystals. In equilibrium the protein concentration remaining in solution will be homogeneous and equal to the saturation concentration. Fig. 6 ▶(*b*) differs from the conditions of Fig. 6 ▶(*a*) in that the dimensionless nucleation rate is higher,  = 27. More crystals are formed, even though the duration of the quench at which Fig. 6 ▶(*b*) is recorded,  = 50, is less than that in Fig. 6 ▶(*a*). The protein in solution has obtained the equilibrium value in between the two rightmost crystals. A noteworthy observation is the development of a depletion zone in the neighborhood of each growing crystal. If the local concentration is reduced sufficiently, then no additional crystals will nucleate in the depletion zone. The size of the depletion zone differs between the two figures; therefore the depletion zone is a function of the nucleation rate .

Fig. 6 ▶(*c*) shows the average number of crystals per drop as a function of time obtained from the simulation and compared with a fit to

The simulation conditions were identical to the conditions of Figs. 6 ▶(*a*) and 6 ▶(*b*): a drop size of 60 µm and two nucleation rates,  = 27 and  = 1. The simulation and fit to equation (3) overlap completely. Equation (3) has two fitting parameters: , the final number of crystals per drop, and , the non­dimensional rate at which crystals form. Fig. 6 ▶(*d*) shows how the final number of crystals per drop varies as a function of drop size for two nucleation rates,  = 27 and  = 1, while Fig. 6 ▶(*e*) shows how the dimensionless rate, , varies with size.

Figs. 6 ▶(*a*) and 6 ▶(*b*), showing the concentration profile inside a supersaturated drop of protein solution during crystallization, are suggestive of a depletion zone in the vicinity of a growing crystal in which the supersaturation is reduced sufficiently such that no new crystals can be nucleated. Let  be the width of this depletion zone and let  be the time interval for which the average number of crystals nucleated in a volume  is one, where  is the spatial dimension. Then, from equation (1) it follows that

which provides one equation relating the depletion zone to the nucleation time. In order for no additional crystals to nucleate in the depletion zone, the protein in solution must be able to diffuse through the depletion zone to the growing crystal, thereby lowering the supersaturation in the depletion zone, in less than the depletion time. This provides a second equation between the depletion zone and nucleation time,

To be self-consistent, we combine equations (4) and (5), which yields

Fig. 6 ▶(*d*) shows the simulated dependence of the number of crystals per drop, , as a function of drop size in one dimension, , for which . Let us examine the curve with the higher nucleation rate,  = 27. For small drops, with a dimensionless drop size of less than ∼9, the number of crystals per drop remains constant at  = 1. ‘Small’ means , *i.e.* the time for the protein to diffuse the entire length of the drop is less than the nucleation time, so that after one crystal has been nucleated, its growth causes a negative feedback suppressing further nucleation throughout the entire drop. As the size of the drop is increased beyond the depletion zone ,  becomes greater than one and the number of crystals per drop grows linearly with drop size. Each nucleation event produces a new depletion zone with just one crystal inside. This process repeats until the entire drop is filled with crystals, each occupying a part of the drop equal to the depletion zone . This scenario predicts that the number of crystals per drop is

The dashed lines in Fig. 6 ▶(*d*) show this behavior; the lines start at the origin and have slope 1/. The ratio of nucleation rates in the two examples shown in Fig. 6 ▶(*d*) is 27, and as , the prediction is that the ratio of the slopes of the dashed lines in Fig. 6 ▶(*d*) is 3, as observed. Furthermore, the width of the depletion zone scales as . Thus for the drop in Fig. 6 ▶(*d*) with a slow nucleation rate  = 1, the width of the depletion zone, manifested by the drop size for which  first becomes greater than one, is predicted to be three times greater than the depletion zone of the drop with the fast nucleation rate  = 27, as observed in Fig. 6 ▶(*d*).

As the drop volume, , is increased from zero, the rate, , of nucleating one crystal in  will increase linearly with drop volume as predicted by equation (1) for Poisson processes,

the behavior seen in Fig. 6 ▶(*e*). However, once the drop volume exceeds the volume of the depletion zone, a crystal will be nucleated somewhere else in the drop; therefore the frequency at which depletion zones are created is

Equations (6) and (9) predict that  in one dimension and that  becomes independent of drop size , as also seen in Fig. 6 ▶(*e*).

The picture that emerges from these simulations and dimensional analyses suggests that nucleation of multiple crystals in a drop is a Poisson process. This is an unexpected result as the nucleation rate is not constant: once the first crystal has nucleated, its growth acts to suppress further nucleation. However, we argue that each nucleation event creates a depletion zone in which it is only possible for one crystal to exist. Therefore, each nucleation event is an independent process. In effect, each drop can be thought of as being partitioned into  smaller independent drops of volume  that nucleate with rate  (Fig. 6 ▶ *f*). This justifies equation (3) and explains why in Fig. 6 ▶(*c*) the number of crystals per drop as a function of time is an exponential, a result indicative of a Poisson process.

The degree to which growing crystals create depletion zones is expected to be greatest in one dimension. For example, in one dimension no protein can be replenished in the gap between two crystals, while in higher dimensions protein will diffuse into the gap between crystals along the directions perpendicular to the line connecting the centers of the crystals. Nevertheless, we expect the same general trends observed in one dimension to carry over to two and three dimensions. In particular, in dimension  we expect that there will be a drop volume , below which only one crystal will be nucleated per drop.

The question of how many crystals will form per drop has been addressed theoretically and experimentally in several studies. Our work is closest in spirit and results to that of Dombrowski *et al.* (2010 ▶) in that we both assume that a single crystal per drop results from a competition between nucleation and growth rates. The differences are that our model explicitly treats spatial concentration variations and assumes that the crystal growth is diffusion limited. In contrast, Dombrowski *et al.* (2010 ▶) assume that the velocity of crystal growth is the rate-limiting step. Then, as the crystal slowly grows, the concentration in the drop remains uniform but decreases with time, eventually shutting off nucleation. In our experiments, we used high supersaturation in order to have a significant probability of nucleating a crystal in drops whose volume is less than a nanolitre. This leads to a fast growth rate in which diffusion of protein from solution to the crystal limits the rate of growth. Experimental measurements of the number of crystals per drop as a function of drop size agree with our model (Maeki *et al.*, 2011 ▶; Akella, 2014 ▶). Another theory (Maeki *et al.*, 2012 ▶) balances the rate of crystal growth with the diffusive flux of protein in order to identify a critical size for obtaining one crystal per drop. Their theory leaves out the key physics of balancing nucleation and growth and disagrees with our model, simulations and experiments (Akella, 2014 ▶).
