# Room-temperature serial crystallography using a kinetically optimized microfluidic device for protein crystallization and on-chip X-ray diffraction. Corrigendum

**DOI:** 10.1107/S205225251501492X

**Published:** 2015-08-14

**Authors:** Michael Heymann, Achini Opathalage, Jennifer L. Wierman, Sathish Akella, Doletha M. E. Szebenyi, Sol M. Gruner, Seth Fraden

**Affiliations:** a Graduate Program in Biophysics and Structural Biology, Brandeis University, 415 South Street, Waltham, MA 02454, USA; b Martin Fisher School of Physics, Brandeis University, 415 South Street, Waltham, MA 02454, USA; c Field of Biophysics, Cornell University, Ithaca, NY 14853, USA; d Cornell High Energy Synchrotron Source (CHESS) and Macromolecular Diffraction Facility at CHESS (MacCHESS), Cornell University, Ithaca, NY 14853, USA; e Department of Physics, Cornell University, Ithaca, NY 14853, USA; f Kavli Institute at Cornell for Nanoscale Science, Cornell University, Ithaca, NY 14853, USA

**Keywords:** protein crystallization, X-ray diffraction, serial crystallography, microfluidic devices

## Abstract

The name of one of the authors in the article by Heymann *et al.* [(2014), *IUCrJ*, **1**, 349–360] is corrected.

## Abstract 2

A correction is made to the article by Heymann *et al.* [(2014), *IUCrJ*, **1**, 349–360].

In the article by Heymann *et al.* (2014 ▸) the name of the second author was given incorrectly. The correct name should be Achini Opathalage as given above.
